# Stroke‐Heart Syndrome: Mechanisms, Temporal Phenotypes, and Diagnostic Reasoning

**DOI:** 10.1002/advs.78548

**Published:** 2026-10-28

**Authors:** Fa Lin, Jian Lyu, Yanci Liu, Zhiyuan Feng, Jie Bao, Wenxiong Song, Bingcheng Zhu, Minghao Liu, Jionghao Xue, Jiadi Weng, Jiani Zhang, Jizong Zhao, Xiaolin Chen, Bin Dong

**Affiliations:** ^1^ Department of Neurosurgery Beijing Tiantan Hospital Capital Medical University Beijing People's Republic of China; ^2^ China National Clinical Research Center for Neurological Diseases Beijing People's Republic of China; ^3^ Department of Neurology Xiyuan Hospital China Academy of Chinese Medical Sciences Beijing People's Republic of China; ^4^ NMPA Key Laboratory for Clinical Research and Evaluation of Traditional Chinese Medicine Beijing People's Republic of China; ^5^ China National Clinical Research Center for Chinese Medicine Cardiology Beijing People's Republic of China; ^6^ Department of Cardiology Beijing Anzhen Hospital Capital Medical University Beijing People's Republic of China; ^7^ Department of Neurology Beijing Tiantan Hospital Capital Medical University Beijing People's Republic of China; ^8^ Senior Department of Cardiology The Sixth Medical Center of PLA General Hospital Beijing People's Republic of China; ^9^ Beijing Chaoyang Hospital Capital Medical University Beijing People's Republic of China; ^10^ Affiliated Hospital of Hebei University Baoding Hebei People's Republic of China

**Keywords:** autonomic dysfunction, brain‐heart axis, diagnostic reasoning, myocardial injury, stroke‐heart syndrome

## Abstract

Stroke‐heart syndrome (SHS) encompasses new or worsened cardiac abnormalities that arise after acute stroke, most often within the first 30 days and with an early peak. Its spectrum includes acute myocardial injury, electrocardiographic changes, arrhythmia, ventricular dysfunction, Takotsubo syndrome, and heart failure, but similar findings may reflect antecedent cardiac disease or systemic illness. This Review defines the accepted clinical boundaries of SHS and integrates central autonomic network disruption, neuroendocrine stress, inflammation, endothelial and microvascular dysfunction, oxygen supply‐demand imbalance, and premorbid substrate into a time‐resolved framework. Eight provisional, non‐mutually‐exclusive phenotypic domains are proposed to organize current evidence without implying validated endotypes. A diagnostic approach based on symptom chronology, prior cardiac data, serial troponin and electrocardiography, rhythm monitoring, echocardiography, selective coronary assessment, and cardiac magnetic resonance is presented to distinguish stroke‐triggered injury from recent myocardial infarction, pre‐existing atrial fibrillation, chronic myocardial injury, and established heart failure. No syndrome‐specific treatment is validated; care should prioritize recognized cardiac emergencies, correction of reversible stressors, and coordinated stroke‐cardiology decision‐making. Prospective studies with documented premorbid status, standardized time points, and blinded adjudication are needed to validate the proposed phenotypes and determine whether they improve outcomes.

## Graphical abstract

Stroke‐heart syndrome describes new or worsened cardiac abnormalities after acute stroke. This Review defines its clinical boundaries, traces autonomic, inflammatory, endothelial, and supply‐demand mechanisms across time, and proposes provisional phenotypes that integrate lesion location, premorbid cardiac substrate, serial biomarkers, electrocardiography, and imaging. The framework supports safer differentiation from primary cardiac disease and more individualized monitoring and management.

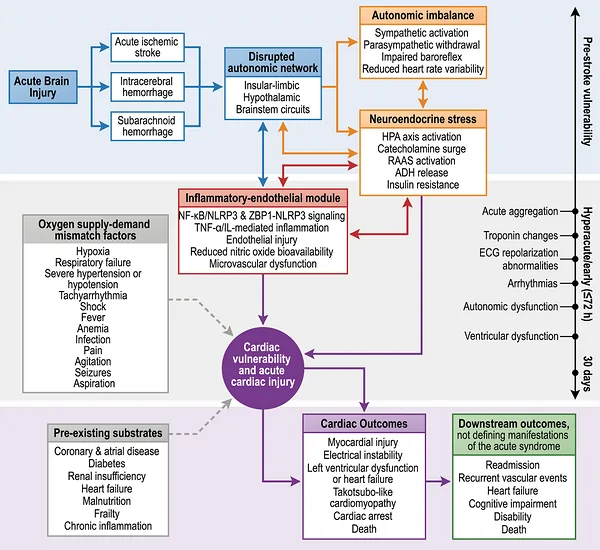

**Abbreviations**
ACS: Acute coronary syndrome
ADH: Antidiuretic hormone
AF: Atrial fibrillation
AFDAS: Atrial fibrillation detected after stroke
APRI: Aspartate aminotransferase‐to‐platelet ratio index
BP: Blood pressure
CMR: Cardiac magnetic resonance
CRP: C‐reactive protein
CRP‐TyG: C‐reactive protein‐triglyceride‐glucose index
ECG: Electrocardiogram
FIB‐4: Fibrosis‐4 index
HF: Heart failure
HPA: Hypothalamic‐pituitary‐adrenal
HR: Heart rate
HRV: Heart‐rate variability
HTN: Hypertension
ICH: Intracerebral hemorrhage
IL: Interleukin
IL‐1β: Interleukin‐1β
IL‐6: Interleukin‐6
LAE: Left‐atrial enlargement
LV: Left ventricular
LVEF: Left ventricular ejection fraction
MI: Myocardial infarction
NF‐κB: Nuclear factor‐κB
NLRP3: NOD‐like receptor protein 3
PRAISE: Prediction of Acute Coronary Syndrome in Acute Ischemic Stroke
QTc: Corrected QT interval
RAAS: Renin‐angiotensin‐aldosterone system
RR: Respiratory rate
SAH: Subarachnoid hemorrhage
SHS: Stroke‐heart syndrome
ST‐T: ST‐segment and T‐wave
TIA: Transient ischemic attack
TNF‐α: Tumor necrosis factor‐α
VT: Ventricular tachycardia
ZBP1: Z‐DNA‐binding protein 1

## 1 Introduction

Stroke‐hearts syndrome (SHS) denotes new cardiac abnormalities, or a documented worsening of premorbid cardiac function, after acute stroke. The proposed acute window extends to 30 days, although manifestations cluster within the first 72 h. The accepted spectrum includes acute myocardial injury, acute coronary syndrome, left ventricular dysfunction or heart failure, Takotsubo syndrome, electrocardiographic repolarization abnormalities, arrhythmias, and sudden cardiac death. In an adjudicated ischemic‐stroke cohort, 19% of patients experienced a serious cardiac adverse event within 3 months and 4.1% died of cardiac causes; the event hazard peaked on days 2–3. Contemporary syntheses estimate that severe cardiac events requiring urgent evaluation or treatment occur in approximately 10%–20% of patients, while overt myocardial infarction, heart failure, Takotsubo syndrome, malignant ventricular arrhythmia, or cardiac arrest occurs in roughly 1%–5%, depending on definitions and ascertainment [1, 2, 3, 4, 5].

The clinical importance of SHS extends beyond the index admission. Early cardiac complications are associated with greater short‐term mortality, functional dependence, and subsequent major adverse cardiovascular events. Cardiac readmission within 30 days and incident heart failure represent measurable health‐system burdens, and observational cohorts link SHS‐coded cardiac complications with later dementia and mortality [6, 7, 8]. These associations do not establish that brain‐triggered myocardial injury causes every downstream event. They show instead that the cardiac phenotype identified after stroke contains prognostic information that should not be dismissed as an incidental laboratory abnormality.

The syndrome remains poorly understood because stroke and cardiac disease share risk factors, premorbid electrocardiograms, biomarkers, and imaging are often unavailable, symptoms may be masked by aphasia or impaired consciousness, and several mechanisms can coexist. Insular and broader central autonomic network injury provides causal plausibility, while emerging work implicates inflammatory and endothelial pathways; nevertheless, there is no validated test that assigns a cardiac abnormality uniquely to the stroke [9, 10, 11, 12, 13]. This Review therefore addresses the gap between a broad descriptive label and clinically disciplined attribution. It first defines the accepted spectrum and exclusions, then integrates neuroanatomical and systemic mechanisms across time, proposes provisional phenotypic domains, and presents a diagnostic framework that keeps antecedent cardiac disease and stroke‐triggered injury in the same differential diagnosis.

## 2 Conceptual Boundaries and the Accepted Clinical Spectrum of Stroke‐Heart Syndrome

The currently accepted SHS spectrum is centered on cardiac abnormalities that are newly detected or demonstrably worsened after stroke. Myocardial injury is its most frequently studied component, usually defined by cardiac troponin above the assay‐specific 99th‐percentile upper reference limit. Elevated troponin is associated with stroke severity, functional outcome, recurrent major vascular events, and mortality, but an isolated value is not a mechanistic diagnosis [14, 15, 16, 17, 18, 19]. The universal definition of myocardial infarction distinguishes myocardial injury from infarction: a rise or fall establishes acute injury, whereas myocardial infarction additionally requires evidence of acute ischemia. In stroke, the differential also includes chronic myocardial injury, renal or structural heart disease, myocarditis, pulmonary embolism, sepsis, Takotsubo syndrome, and neurogenic injury [20, 21, 22, 23].

Electrical manifestations include ST‐T abnormalities, QTc prolongation, conduction disturbance, premature beats, atrial arrhythmia, and ventricular arrhythmia [24, 25, 26]. Their prevalence and prognostic value depend on baseline tracings, medication exposure, electrolyte status, stroke severity, and monitoring intensity. Composite electrocardiographic scores and heart‐rate turbulence may refine risk estimates, but neither identifies a unique SHS mechanism [27, 28]. Their most reliable clinical role is to trigger appropriately scaled serial assessment rather than to assign etiology from a single tracing.

Atrial fibrillation illustrates the bidirectionality of brain‐heart disease. Rhythm first documented after stroke may represent previously occult atrial fibrillation that caused the event, high‐burden atrial fibrillation newly identified on the admission electrocardiogram, an arrhythmia unmasked by monitoring, or a rhythm disturbance facilitated by acute autonomic injury [29, 30, 31, 32, 33, 34]. Prolonged monitoring increases detection, but detection time is not biological onset [35]. Accordingly, the neutral term atrial fibrillation detected after stroke (AFDAS) is preferable until prior recordings, arrhythmia burden, atrial substrate, lesion features, and follow‐up clarify the most likely direction.

Acute ventricular dysfunction and Takotsubo syndrome provide a more visible neurocardiac phenotype. Transient regional or global dysfunction can accompany ischemic stroke, intracerebral hemorrhage, and particularly subarachnoid hemorrhage [36, 37, 38, 39]. Insular injury and sympathetic activation support a neurogenic contribution in selected cases, but Takotsubo syndrome, myocarditis, secondary myocardial infarction, sepsis‐related cardiomyopathy, and coronary disease can overlap [40, 41]. The SHS framework should therefore promote tissue‐ and time‐informed adjudication rather than treat the absence of a culprit coronary lesion as proof of neurogenic injury.

Hemorrhagic stroke and contemporary reperfusion care broaden the accepted clinical context while also exposing heterogeneity. Intracerebral hemorrhage and subarachnoid hemorrhage can generate abrupt intracranial‐pressure changes, catecholamine release, electrical instability, and ventricular dysfunction, while simultaneously constraining antithrombotic and invasive cardiac strategies [42, 43, 44]. Endovascular thrombectomy adds large‐vessel occlusion, infarct burden, anesthesia or sedation, hemodynamic variability, contrast exposure, and reperfusion injury to the substrate [45, 46]. Recent nutritional, inflammatory‐metabolic, and fibrosis‐index models are useful for risk stratification, but their associations should not be interpreted as proof that these indices cause SHS [47, 48, 49].

This Review proposes a narrower operational boundary. SHS should be considered when the timing, trajectory, anatomical context, or physiological pattern supports cardiac injury that was triggered, amplified, or unmasked by acute cerebral injury. It should not be assigned to a recent myocardial infarction that clearly predates or plausibly causes the stroke, stable chronic troponin elevation, known atrial fibrillation or heart failure without demonstrable change, or cardiac injury better explained by a non‐neurological systemic illness [50]. Long‐term readmission, recurrent vascular events, cognitive decline, and death are important outcomes for prognosis and validation; they are not defining manifestations of the acute syndrome. When evidence remains incomplete, the appropriate classification is probable, possible, mixed, or temporally indeterminate rather than a forced binary label.

## 3 Biological Architecture of Stroke‐Heart Syndrome

### 3.1 Central Autonomic Network

The central autonomic network provides the most direct route from focal cerebral injury to cardiac physiology. It includes the insula, anterior cingulate cortex, amygdala, hypothalamus, prefrontal regions, brainstem nuclei, and descending spinal autonomic pathways. Experimental insular stimulation alters chronotropy and can provoke lethal arrhythmia, while human stimulation and lesion studies link insular injury with lateralized autonomic responses, impaired heart‐rate variability, arrhythmia, and myocardial injury [51, 52, 53, 54]. Baroreflex impairment and network‐disconnection analyses further indicate that risk is not reducible to one cortical region [55, 56].

An insula‐only model is therefore insufficient. The anterior cingulate participates in autonomic salience, the amygdala integrates threat and sympathetic output, the hypothalamus coordinates neuroendocrine efferents, and brainstem nuclei govern baroreflex, chemoreflex, respiratory‐cardiac coupling, and parasympathetic output. Lesion side, subregion, depth, edema, diaschisis, and disconnection vary substantially, as do premorbid autonomic tone and cardiac reserve. Central autonomic network involvement should therefore modify the prior probability of neurogenic injury, not determine the diagnosis on its own.

This network model also explains why laterality findings are inconsistent. Right‐insular injury has often been associated with sympathetic dominance, reduced heart‐rate variability, and arrhythmia, while left‐insular stimulation has been linked to bradycardic or depressor responses. Clinical lesions, however, vary in subregion, vascular territory, depth, edema, and connected‐network disruption. Medication, atrial disease, coronary reserve, sleep‐disordered breathing, diabetes, renal function, and frailty further modify the observed response. Lesion‐informed neurocardiology should therefore integrate atlas‐based network involvement with serial telemetry and biomarker trajectories instead of using lesion side as a deterministic rule.

### 3.2 Autonomic and Neuroendocrine Translation

After central autonomic disruption, sympathetic activation increases heart rate, blood pressure, contractility, wall stress, coronary microvascular tone, and electrical excitability. Parasympathetic withdrawal reduces vagal buffering and baroreflex control. These changes interact with hypoxemia, pain, agitation, fever, seizures, aspiration, blood‐pressure extremes, and vasoactive treatment [57, 58, 59, 60, 61]. Catecholamine excess may promote calcium overload, oxidative stress, mitochondrial dysfunction, contraction‐band necrosis, microvascular constriction, and transient ventricular dysfunction. It is a major pathway, but cannot explain stable chronic biomarker elevation or every mild troponin rise.

Heart‐rate variability, heart‐rate turbulence, baroreflex sensitivity, blood‐pressure variability, and cardiorespiratory coupling are attractive because they measure processes close to the proposed mechanism [62, 63, 64, 65]. They are also sensitive to age, atrial fibrillation, ectopy, respiration, sleep, sedation, ventilation, fever, and recording quality. At present, they are translational biomarkers, not diagnostic criteria. Their value depends on standardized acquisition, transparent preprocessing, and demonstration of incremental information beyond stroke severity and pre‐existing cardiac disease.

Neuroendocrine activation extends the pathway beyond autonomic efferents. Cortisol release, renin‐angiotensin‐aldosterone signaling, vasopressin, insulin resistance, and inflammatory activation can alter myocardial oxygen demand, vascular tone, volume status, endothelial permeability, and arrhythmic susceptibility. The same signal can therefore yield different cardiac phenotypes in a young patient with preserved reserve and in an older patient with hypertrophy, coronary stenosis, atrial remodeling, renal dysfunction, or chronic inflammation. This interaction between acute neural stress and premorbid substrate is central to the heterogeneity of SHS.

Catecholamine‐mediated injury also overlaps with, but is not synonymous with, Takotsubo syndrome. Calcium overload and contraction‐band necrosis may occur without the classic ballooning pattern, whereas ventricular dysfunction after stroke may arise from coronary ischemia, myocarditis, sepsis, or volume and pressure loading. Conversely, modest troponin release can occur without a detectable reduction in ejection fraction. Mechanistic studies should therefore measure autonomic activity, myocardial tissue, coronary anatomy, and systemic stress within the same time window rather than infer one pathway from a single downstream marker. Figure 1 provides an integrated map of the autonomic, neuroendocrine, inflammatory, endothelial, microvascular, and supply‐demand pathways developed throughout this section.

**FIGURE 1:**
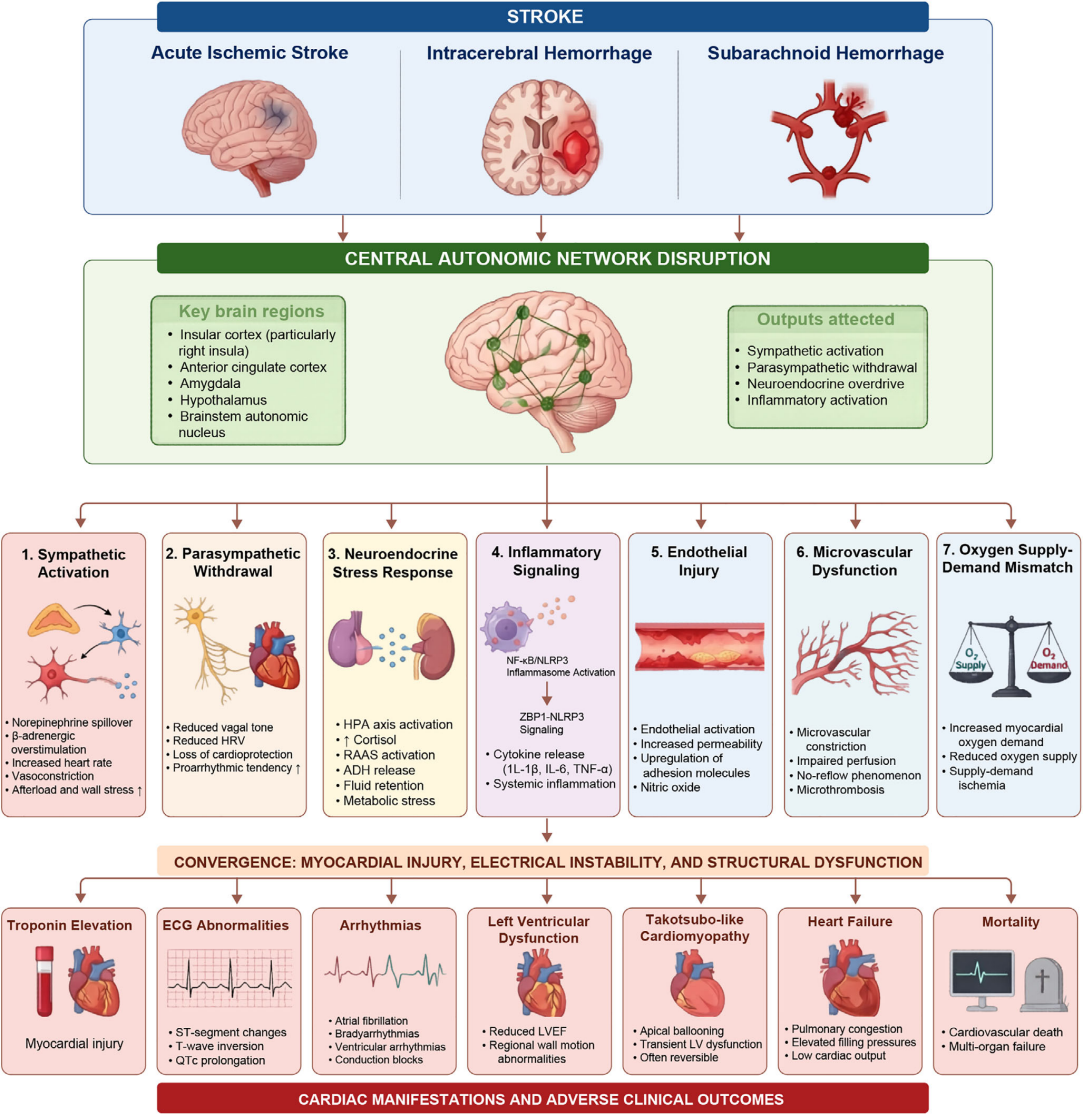
Mechanistic cascade from acute stroke to cardiac manifestations. Ischemic stroke, intracerebral hemorrhage, and subarachnoid hemorrhage can disrupt insular‐limbic, hypothalamic, and brainstem autonomic networks. Sympathetic activation, parasympathetic withdrawal, neuroendocrine stress, NF‐κB/NLRP3 and ZBP1‐NLRP3 signaling, endothelial injury, microvascular dysfunction, and oxygen supply‐demand mismatch may converge on myocardial injury, electrical instability, ventricular dysfunction, Takotsubo‐like cardiomyopathy, heart failure, and death. Abbreviations: ADH, antidiuretic hormone; ECG, electrocardiogram; HPA, hypothalamic‐pituitary‐adrenal; HRV, heart‐rate variability; IL, interleukin; LVEF, left ventricular ejection fraction; NF‐κB, nuclear factor‐κB; NLRP3, NOD‐like receptor protein 3; RAAS, renin‐angiotensin‐aldosterone system; SHS, stroke‐heart syndrome; TNF‐α, tumor necrosis factor‐α; ZBP1, Z‐DNA‐binding protein 1.

### 3.3 Inflammatory and Endothelial Signaling

Acute cerebral injury activates the hypothalamic‐pituitary‐adrenal axis, circulating catecholamines, leukocyte mobilization, cytokine release, and endothelial perturbation. Early human studies linked troponin release with cortisol, tumor necrosis factor‐α, and sympathoadrenal activation, and later cohorts associated inflammatory and cardiovascular biomarkers with mortality [66, 67, 68, 69]. These findings support interaction between autonomic and immune pathways, but circulating inflammation can also reflect infarct volume, infection, frailty, or chronic vascular disease.

The nuclear factor‐κB (NF‐κB)/NOD‐like receptor protein 3 (NLRP3) axis links inflammatory transcription, inflammasome activation, endothelial injury, and microvascular dysfunction. Experimental work suggests sustained endothelial NLRP3 activation after cerebral ischemia and identifies Z‐DNA‐binding protein 1 (ZBP1)‐NLRP3 signaling as another candidate pathway [12, 70, 71]. Endothelial dysfunction can reduce nitric oxide bioavailability, increase permeability and leukocyte adhesion, promote microvascular constriction, and lower the myocardial ischemic threshold [72]. These pathways are mechanistically plausible; no inflammasome inhibitor or endothelial‐protective strategy has yet been shown to improve clinical SHS outcomes.

Endothelium is a useful integrator because it connects neural stress, circulating inflammation, coronary microcirculation, and tissue injury. A microvascular phenotype can impair perfusion or increase permeability without producing one obstructive epicardial lesion, which is consistent with the mixed coronary findings in troponin‐positive stroke cohorts. This does not establish that endothelial dysfunction is the dominant cause in an individual patient. Future translational studies require timed target‐engagement markers, cell‐specific evidence, and a safety framework that accounts for infection, repair, and hemorrhagic risk.

The inflammatory literature also illustrates the distinction between association and intervention. Inflammatory proteins may mediate myocardial injury, mark a large infarct, identify chronic vascular risk, or reflect intercurrent infection. An experimental pathway can be biologically real without being the most important determinant of a patient's troponin elevation. Mechanistic enrichment should therefore combine stroke subtype and lesion location with serial inflammation, endothelial biomarkers, cardiac imaging, and coronary adjudication before a pathway is advanced as a therapeutic target.

### 3.4 Supply‐Demand Imbalance, Coronary Overlap, and Substrate

Myocardial oxygen supply‐demand imbalance links neurogenic stress with conventional cardiac pathophysiology. Hypoxemia, respiratory failure, severe hypertension or hypotension, tachyarrhythmia, shock, fever, anemia, and infection can produce acute injury in a patient with limited coronary or microvascular reserve. Coronary angiography demonstrates the resulting heterogeneity: some troponin‐positive stroke patients have clinically important coronary disease, whereas many lack a culprit lesion [73, 74, 75, 76]. The appropriate inference is selective coronary evaluation, not automatic attribution to either plaque rupture or the brain.

Cardiac magnetic resonance (CMR) adds tissue characterization when routine tests remain ambiguous. Infarction, myocarditis‐like injury, Takotsubo patterns, focal scar, diffuse myocardial disease, or no substantial tissue abnormality carry different implications [77, 78, 79]. Coronary‐territory late gadolinium enhancement with edema supports recent infarction; old enhancement without edema favors prior scar; transient noncoronary dysfunction or edema without a coronary pattern supports, but does not prove, stress‐related injury. Feasibility and selection bias limit CMR during unstable acute stroke.

Finally, the injured brain acts on a heterogeneous substrate. Coronary and atrial disease, diabetes, renal dysfunction, heart failure, malnutrition, frailty, chronic inflammation, and reduced metabolic reserve alter the threshold at which autonomic or systemic stress becomes myocardial injury. Diabetes‐ and thrombectomy‐focused studies support this vulnerability concept, while prolonged cardiac and vascular imaging can uncover occult atrial or aortic sources [80, 81, 82]. Substrate variables should be modeled as modifiers and alternative explanations rather than mislabeled as brain‐derived mechanisms.

Atrial substrate deserves particular attention because it can lie on both sides of the causal sequence. Atrial enlargement, fibrosis, ectopy, inflammation, and autonomic imbalance predispose to embolic stroke and to rhythm instability during acute illness. Some AFDAs represent occult pre‐existing disease uncovered by monitoring; some may reflect a vulnerable atrium destabilized by stroke; and some are detected because the patient enters an unusually intensive surveillance environment. Rhythm burden, detection method, atrial phenotype, embolic pattern, and recurrence risk are therefore more informative than the label “new‐onset.”

Frailty and malnutrition broaden substrate beyond conventional cardiovascular diagnoses. Reduced muscle and cardiac reserve, impaired immune regulation, autonomic blunting, micronutrient deficiency, and susceptibility to infection or hypoxia can magnify an otherwise tolerated stress response. Composite indices such as CRP‐TyG and FIB‐4 may summarize aspects of inflammatory, metabolic, or hepatic vulnerability, but their predictive associations are population‐ and treatment‐context dependent. Their immediate value is risk description; causal or treatment claims require dedicated validation.

## 4 Temporal Evolution and Provisional Phenotypic Domains

SHS is best measured as a trajectory rather than an admission state. Premorbid cardiac and systemic substrates define reserve before stroke. During the hyperacute phase, central autonomic disruption, neuroendocrine stress, respiratory compromise, blood‐pressure extremes, and inflammation create the first wave of risk. During the first week, troponin change, repolarization abnormalities, arrhythmia, autonomic dysfunction, and ventricular dysfunction may evolve. Subsequent heart failure, cardiac readmission, recurrent vascular events, cognitive impairment, disability, and death are downstream outcomes rather than criteria for the acute syndrome. Elevated troponin is associated with recurrent major vascular events and death, although individual recurrent‐stroke associations are less consistent [83, 84].

Eight provisional and non‐mutually‐exclusive phenotypic domains can organize this trajectory: autonomic‐dominant, demand‐ischemia, inflammatory‐endothelial, Takotsubo‐like, coronary‐overlap, arrhythmia‐dominant, hemorrhagic‐stress, and systemic‐vulnerability. These labels deliberately mix mechanism, phenotype, clinical context, and modifier because the available evidence cannot yet support biologically pure endotypes. A patient may occupy several domains, and the dominant probability may change as serial data accrue. The framework is therefore a testable organizational model, not a validated classification system.

Outcome evidence should be interpreted at the component level. Autonomic disturbance, myocardial injury, electrical instability, ventricular dysfunction, hemorrhagic‐stroke cardiac injury, and systemic vulnerability markers have each been associated with adverse outcomes in selected cohorts. These studies support biological plausibility and prognostic relevance, but they do not prove that the proposed composite domains are causally distinct or independently predictive. Prospective assignment, blinded cardiac adjudication, and external validation are required before the domains can guide syndrome‐specific treatment. Figure 2 maps the temporal framework, and Table 1 summarizes the proposed phenotypic domains and their evidentiary limits.

**FIGURE 2:**
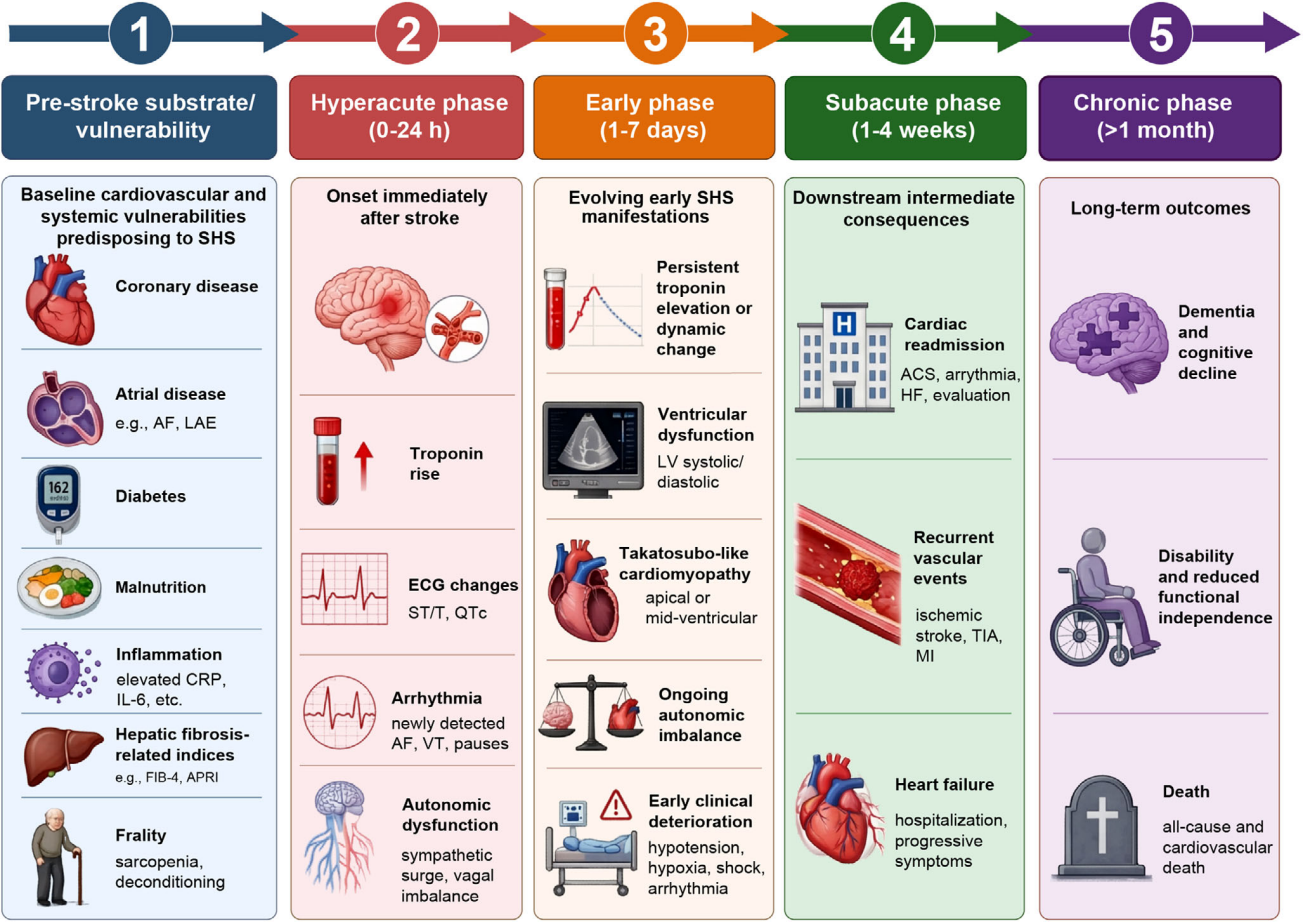
Temporal framework for SHS manifestations and outcomes. Premorbid cardiac and systemic vulnerability precedes stroke. Hyperacute and early findings include troponin change, electrocardiographic abnormalities, arrhythmia, autonomic disturbance, and ventricular dysfunction. Readmission, recurrent vascular events, heart failure, cognitive impairment, disability, and death are downstream outcomes, not defining acute manifestations. Abbreviations: ACS, acute coronary syndrome; AF, atrial fibrillation; APRI, aspartate aminotransferase‐to‐platelet ratio index; CRP, C‐reactive protein; ECG, electrocardiogram; FIB‐4, fibrosis‐4 index; HF, heart failure; IL, interleukin; LAE, left‐atrial enlargement; LV, left ventricular; MI, myocardial infarction; SHS, stroke‐heart syndrome; TIA, transient ischemic attack; VT, ventricular tachycardia.

**TABLE 1:** Provisional phenotypic domains in stroke‐heart syndrome.

Phenotypic domain	Defining logic	Typical pattern	Best discriminators	Representative component evidence and reported associations
Autonomic‐dominant	Central autonomic network injury promotes sympathetic activation and vagal withdrawal	Insular/brainstem involvement; labile pressure; QTc prolongation; arrhythmia	Lesion mapping; telemetry; heart‐rate variability/turbulence; baroreflex metrics	Lesion and autonomic measures associate with myocardial injury, complications, and functional outcome [11, 52, 53, 54, 55, 56, 57, 58, 59, 60, 61, 62, 63, 64, 65].
Demand‐ischemia	Acute physiological stress exceeds myocardial oxygen supply or reserve	Hypoxemia; shock; pressure extremes; tachyarrhythmia; fever; anemia; infection	Serial troponin; ischemic evidence; hemodynamics; oxygenation; echocardiography	Acute injury and demand triggers are associated with mortality and adverse outcome [1, 4, 20, 21, 22, 74].
Inflammatory‐endothelial	Neuroimmune and endothelial activation may promote microvascular injury	High inflammatory burden; severe/reperfused stroke; no single culprit lesion	Inflammatory/endothelial markers; CMR; coronary assessment	Inflammatory biomarkers associate with mortality; molecular pathways remain experimental [12, 66, 67, 68, 69, 70, 71, 72].
Takotsubo‐like	Catecholamine stress accompanies transient ventricular stunning	Subarachnoid hemorrhage or strategic stroke; apical/mid‐ventricular dysfunction	Echocardiography; CMR; serial ECG/troponin; coronary exclusion	Clinical series document transient dysfunction and hemodynamic complications [36, 37, 38, 39, 40, 41, 85].
Coronary‐overlap	Stroke coexists with or precipitates clinically important coronary ischemia	Ischemic symptoms/ECG evolution; high absolute troponin; coronary‐territory dysfunction	Coronary imaging; CMR; serial ECG/troponin; echocardiography.	Angiographic studies identify a clinically important but selected culprit‐lesion subgroup [20, 73, 75, 76].
Arrhythmia‐dominant	Atrial or ventricular instability is triggered or unmasked after stroke	AFDAS; brady‐tachy pattern; ventricular arrhythmia; conduction disturbance	Prior tracings/device data; burden and timing; telemetry; atrial substrate	Detected atrial fibrillation and electrical abnormalities associate with recurrence and death [24, 25, 26, 27, 28, 29, 30, 31, 32, 33, 34, 35].
Hemorrhagic‐stress	Intracerebral or subarachnoid hemorrhage produces intense neurocritical stress	QTc change; troponin rise; arrhythmia; stress cardiomyopathy; instability	Hemorrhage trajectory; serial ECG/troponin; echocardiography; hemodynamics	Dynamic injury and early cardiac complications associate with mortality [23, 38, 39, 42, 43, 44].
Systemic‐vulnerability	Limited physiological reserve amplifies cardiac injury during acute stress	Frailty; malnutrition; diabetes; renal dysfunction; inflammatory‐metabolic indices	Frailty/nutritional measures; renal function; CRP‐TyG; FIB‐4	Selected endovascular cohorts report prognostic associations; causality is unestablished [47, 49, 80].

*Note*: The domains are overlapping hypotheses, not validated endotypes. Citations support component phenotypes and reported associations; they do not validate each composite domain as a causal class. Abbreviations: AFDAS, atrial fibrillation detected after stroke; CMR, cardiac magnetic resonance; CRP‐TyG, C‐reactive protein‐triglyceride‐glucose index; FIB‐4, fibrosis‐4 index.

## 5 Diagnostic Workflow and Temporal Adjudication

### 5.1 From an Abnormal Cardiac Test to Mechanistic Probability

Evaluation should begin with the principle that an abnormal troponin or electrocardiogram is a signal, not a diagnosis. The 2026 American Heart Association/American Stroke Association guideline recommends baseline electrocardiography and cardiac troponin during the initial evaluation of acute ischemic stroke; neither test should delay intravenous thrombolysis or endovascular treatment [86]. A normal initial assessment supports routine stroke‐unit monitoring with repeat testing for clinical change. Elevated troponin, a high‐risk electrocardiographic abnormality, cardiac symptoms, or hemodynamic instability warrants serial, phenotype‐directed assessment and coordinated neurological and cardiological review [21, 87].

Serial troponin distinguishes acute from stable chronic myocardial injury, but a rise or fall does not identify the cause. Acute myocardial infarction requires acute injury plus ischemic evidence from symptoms or signs, new ischemic electrocardiographic changes or pathological Q waves, acute coronary pathology, or new loss of viable myocardium or a coronary‐territory wall‐motion abnormality [20]. Potential demand triggers, including hypoxemia, shock, pressure extremes, tachyarrhythmia, anemia, fever, and infection, should be recorded contemporaneously. Absence of chest pain is weak negative evidence in a patient with aphasia, neglect, sedation, delirium, or impaired consciousness.

Echocardiography is indicated when heart failure, shock, embolic source, stress cardiomyopathy, new ischemic change, or substantial troponin dynamics are suspected. Selective coronary computed tomography angiography or invasive angiography is appropriate when integrated symptoms, electrocardiography, absolute and serial troponin, regional function, coronary history, and neurological bleeding risk leave a meaningful probability of acute coronary disease [73, 75, 76]. CMR can adjudicate tissue biology when coronary and echocardiographic findings do not resolve the diagnosis [77, 78, 79]. Figure 3 presents the practical diagnostic workflow; Table 2 complements it with temporal and mechanistic discriminators.

**FIGURE 3:**
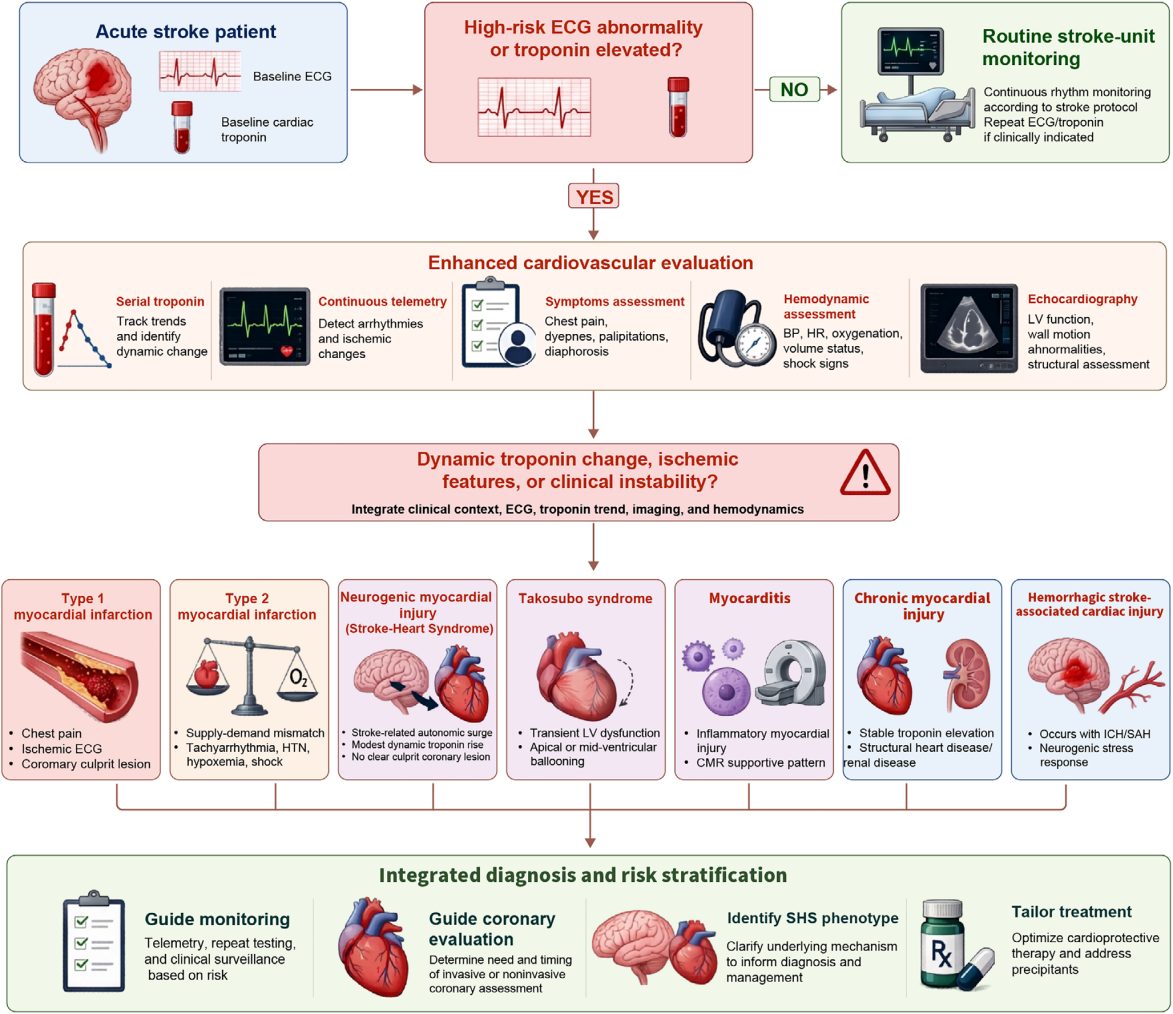
Diagnostic workflow for cardiac abnormalities after acute stroke. Baseline electrocardiography and cardiac troponin support initial triage but should not delay reperfusion treatment. A high‐risk electrocardiographic abnormality, elevated troponin, symptoms, or instability prompts serial biomarkers, telemetry, hemodynamic assessment, and echocardiography. Dynamic troponin identifies acute injury but does not distinguish type 1 myocardial infarction, type 2 myocardial infarction, neurogenic injury, Takotsubo syndrome, myocarditis, or hemorrhagic‐stroke‐associated injury. Absence of a culprit lesion does not prove a neurogenic mechanism. Selective coronary assessment, CMR, and specialist review are used when clinically indicated [20, 21, 73, 75, 76, 77, 78, 79, 86, 87]. Abbreviations: BP, blood pressure; CMR, cardiac magnetic resonance; ECG, electrocardiogram; HR, heart rate; HTN, hypertension; ICH, intracerebral hemorrhage; LV, left ventricular; SAH, subarachnoid hemorrhage; SHS, stroke‐heart syndrome.

**TABLE 2:** Temporal and mechanistic differential diagnosis after acute stroke.

Condition	Temporal and troponin clues	ECG, imaging, or coronary clues	Evidence favoring antecedent vs. stroke‐associated disease	Clinical implication
Type 1 myocardial infarction	Acute rise/fall; cardiac symptoms may precede or be masked by stroke	New ischemic ECG change; coronary‐territory wall‐motion loss; culprit lesion or coronary‐pattern CMR	Pre‐stroke pain/dyspnea/syncope or coronary pathology favors antecedent/causal MI; timing may remain indeterminate	Urgent cardiology/coronary pathway balanced against intracranial bleeding and reperfusion priorities
Type 2 myocardial infarction	Acute injury plus evidence of ischemia during supply‐demand imbalance	Ischemic change without acute atherothrombosis; global or mixed dysfunction may occur	Hypoxemia, shock, tachyarrhythmia, anemia, pressure extremes, or infection after stroke supports secondary ischemia	Treat the precipitant, monitor trajectory, and investigate persistent ischemic evidence
Probable neurogenic myocardial injury	New injury follows stroke; often modest, but no universal magnitude or delta is diagnostic	QTc/repolarization change, autonomic instability, noncoronary dysfunction; no single pathognomonic pattern	Strategic central autonomic‐network lesion and close temporal linkage support; competing causes must be excluded	Telemetry, supportive care, serial reassessment, and probability‐based documentation
Takotsubo syndrome	Acute biomarker change with transient ventricular dysfunction	Apical, mid‐ventricular, basal, or focal pattern; CMR supportive; culprit lesion absent	New dysfunction after severe neurological stress and interval recovery support a stroke‐associated trigger	Supportive heart‐failure care and coronary exclusion tailored to neurological risk
Myocarditis	Variable acute injury; systemic prodrome may precede stroke	Nonischemic edema or late gadolinium enhancement; inflammatory context	Pre‐stroke infection/symptoms and nonischemic CMR favor independent myocarditis	CMR and cardiology‐guided evaluation; avoid neurogenic attribution by exclusion alone
Chronic myocardial injury	Stable elevation or prior similar value	Chronic structural disease, renal disease, or established heart failure	Clearly predates stroke and lacks demonstrable worsening.	Do not classify as SHS; manage the underlying chronic condition
Atrial fibrillation detected after stroke	Detection time is not biological onset; report burden and monitoring method	Prior ECG/device data; atrial size/fibrosis; ectopy; embolic pattern	Admission‐ECG/high‐burden or prior evidence favors antecedent AF; low burden after strategic stroke may be mixed	Use neutral AFDAS terminology until longitudinal evidence supports attribution
Heart failure or ventricular dysfunction	Compare symptoms, therapy, natriuretic peptides, and function with baseline	Chronic remodeling/scar favors antecedent disease; new noncoronary dysfunction with recovery supports acute stress	Classify as known HF, decompensated known/occult HF, probable de novo dysfunction, or indeterminate	Treat congestion/instability; repeat imaging and revise temporal classification

*Note*: A rise or fall in cardiac troponin indicates acute myocardial injury but does not establish its mechanism. Type 2 myocardial infarction requires acute injury plus evidence of ischemia attributable to oxygen supply‐demand imbalance. When chronology remains uncertain, temporally indeterminate or overlap classification is preferred. Abbreviations: AF, atrial fibrillation; AFDAS, atrial fibrillation detected after stroke; CMR, cardiac magnetic resonance; ECG, electrocardiogram; HF, heart failure; SHS, stroke‐heart syndrome.

### 5.2 Stroke‐Triggered Injury vs. Antecedent Cardiac Disease

Directionality should be reconstructed before applying the SHS label. The sequence of neurological and cardiac symptoms should be assembled from the patient, witnesses, emergency medical services, prior electrocardiograms, troponin measurements, echocardiograms, rhythm‐device data, heart‐failure diagnoses, medication changes, and recent coronary procedures. Chest pain, dyspnea, syncope, or instability that clearly precedes the neurological deficit favors antecedent cardiac disease; onset after a well‐defined stroke, coupled with a compatible lesion and evolving autonomic or ventricular phenotype, increases the probability of stroke‐triggered injury. Simultaneous onset or absent baseline data should be documented as temporally indeterminate or coronary‐cerebral overlap.

For myocardial injury, absolute troponin concentration, direction and magnitude of change, ischemic electrocardiographic evolution, wall‐motion territory, and coronary or CMR findings should be interpreted together. A coronary culprit or coronary‐pattern enhancement with edema supports recent myocardial infarction. Transient noncoronary dysfunction or edema without a coronary distribution supports a stress‐related mechanism, but does not by itself prove that the stroke caused the injury. Stable elevation with prior similar values, chronic kidney disease, or established structural disease favors chronic myocardial injury.

For rhythm, three clinically useful states are known atrial fibrillation before stroke, atrial fibrillation newly identified on the admission electrocardiogram, and AFDAS during short‐ or long‐term monitoring. Admission‐electrocardiogram detection often indicates a higher burden and should usually be managed as pre‐existing substrate. Prior device evidence, substantial burden, left‐atrial enlargement or fibrosis, atrial ectopy, high natriuretic peptides, and an embolic imaging pattern favor antecedent atrial disease. Low‐burden paroxysms after strategic autonomic‐network injury support a neurogenic contribution, but no single feature determines biological onset [29, 30, 31, 32, 33, 34, 35].

Heart failure should be classified as known heart failure, acute decompensation of known or occult heart failure, or probable de novo ventricular dysfunction after stroke. Prior symptoms, diuretic or guideline‐directed therapy, natriuretic‐peptide trends, chamber remodeling, valvular disease, and earlier imaging are critical. New congestion or dysfunction after stroke and interval recovery support, but do not prove, a neurogenic or Takotsubo mechanism; chronic remodeling or persistent coronary‐territory scar favors antecedent disease. Natriuretic peptides or reduced ejection fraction alone cannot establish onset. Classification should be updated after serial reassessment rather than fixed at admission.

## 6 Stroke Subtype and Treatment Context

Most SHS evidence derives from acute ischemic stroke, yet lesion burden, vascular territory, mechanism, and reperfusion context differ. A minor non‐insular infarct, a large right‐insular infarct, and an anesthetized patient undergoing thrombectomy create different pretest probabilities and competing stressors. Reperfusion can reduce cerebral injury while introducing hemodynamic variation, contrast exposure, hemorrhagic‐transformation risk, and intensive‐care confounding [88, 89]. Biomarker prediction models in endovascular cohorts require external validation and should not be mistaken for causal models.

Intracerebral hemorrhage produces a related but distinct neurocardiac setting. Abrupt bleeding, mass effect, intracranial‐pressure change, sympathetic activation, and systemic stress can produce electrical and myocardial abnormalities, while hematoma expansion risk limits antithrombotic escalation [90]. Troponin elevation or dynamic change is prognostically important and warrants evaluation, but is not a default indication for a coronary treatment pathway. Serial biomarkers, electrocardiography, echocardiography, hemodynamics, and neurological stability must be integrated [23, 42, 43, 44].

Subarachnoid hemorrhage remains the classical model of neurogenic stunned myocardium and Takotsubo syndrome. QTc prolongation, arrhythmia, ventricular dysfunction, pulmonary edema, and hemodynamic instability can compromise cerebral perfusion and aneurysm treatment [38, 39, 85, 91, 92, 93]. These observations support brain‐to‐heart causality in selected patients but should not be transferred uncritically to ischemic stroke, where the trigger, intracranial dynamics, age distribution, and background cardiac disease differ.

## 7 Management

No syndrome‐specific drug or procedure has been validated for SHS. Management should therefore identify the cardiac phenotype, treat established emergencies according to established cardiac and stroke practice, correct reversible physiological stressors, and balance coronary benefit against cerebral bleeding or perfusion risk. The prospective PRAISE study reinforces that dynamic troponin alone does not identify type 1 myocardial infarction; electrocardiographic, echocardiographic, clinical, and absolute biomarker evidence must be integrated [94].

Hypoxemia, respiratory failure, severe hypertension or hypotension, fever, anemia, infection, pain, agitation, tachyarrhythmia, shock, and volume disturbance should be sought and corrected. Telemetry, repeat electrocardiography and troponin, echocardiography, cardiology consultation, and coronary or CMR evaluation should be scaled to the evolving risk profile. β‐blockade or autonomic modulation may be appropriate for standard cardiac indications in selected patients, but observational associations do not justify routine SHS‐specific use [95]. Experimental anti‐inflammatory, endothelial, or targeted delivery approaches remain preclinical and should not be presented as established care [96].

## 8 Research Priorities

The first research priority is a common data model. Cohorts should document premorbid cardiac status, stroke onset and lesion topology, serial cardiac biomarkers, electrocardiography and rhythm burden, hemodynamics, respiratory and infectious stressors, echocardiography, coronary assessment, CMR, treatment exposure, and outcomes at harmonized time points. Experimental and non‐human‐primate models can then test whether neural, autonomic, endothelial, and myocardial signals identified in patients are causally linked [97, 98, 99, 100, 101, 102, 103].

The second priority is phenotype truth. Prediction models trained on an undefined composite will automate the same ambiguity that limits current studies. Myocardial injury, infarction, Takotsubo syndrome, atrial fibrillation, ventricular dysfunction, heart failure, and cardiac death should be separately adjudicated. Models should report acquisition windows, missingness, calibration, discrimination, external validation, clinical utility, and subgroup performance in accordance with contemporary prediction‐model standards [104, 105, 106].

The third priority is causal and equitable validation. Target‐trial emulation, mediation analysis, trajectory modeling, and prespecified causal diagrams can distinguish markers from mediators and modifiers [107, 108, 109]. Cluster‐derived phenotypes and hierarchical analyses are hypothesis‐generating until they are prospectively assigned and externally reproduced [109]. Minimum‐resource pathways based on electrocardiography, serial troponin, telemetry, clinical stressors, and echocardiography should be evaluated alongside advanced pathways using CMR, coronary imaging, lesion connectomics, and autonomic or endothelial biomarkers.

The proposed phenotypic domains should be judged by three questions: can they be assigned reproducibly, do they add prognostic information beyond stroke severity and baseline cardiac disease, and do they change a decision that improves patient‐centered outcomes? Until those criteria are met, the most defensible use of SHS is as a structured framework for probability‐based diagnostic reasoning and prospective study design.

## 9 Conclusions

This Review addresses the gap between recognition of post‐stroke cardiac abnormalities and reliable attribution of their timing and mechanism. The revised framework separates the accepted acute spectrum from antecedent or competing cardiac disease, treats long‐term events as outcomes rather than diagnostic criteria, and organizes heterogeneous presentations into eight provisional, overlapping phenotypic domains. Central autonomic disruption, neuroendocrine stress, inflammatory‐endothelial signaling, oxygen supply‐demand imbalance, coronary disease, and systemic substrate are complementary probabilities, not mutually exclusive explanations.

The practical advance is temporal adjudication. Prior cardiac data, event chronology, serial troponin and electrocardiography, rhythm burden, echocardiography, selective coronary imaging, and CMR should be combined to distinguish stroke‐triggered injury from recent myocardial infarction, pre‐existing atrial fibrillation, chronic injury, and established heart failure. The remaining knowledge gap is prospective validation: cohorts with documented premorbid status, standardized sampling, blinded cardiac adjudication, and external replication are required to determine whether the proposed domains improve diagnosis, prognosis, or care.

## Author Contributions

**Fa Lin**, **Jian Lyu**, **Jizong Zhao**, **Xiaolin Chen**, and **Bin Dong**: conceptualization. **Fa Lin** and **Jian Lyu**: writing – original draft. **Yanci Liu**, **Zhiyuan Feng**, **Jie Bao**, **Wenxiong Song**, **Bingcheng Zhu**, **Minghao Liu**, **Jionghao Xue**, **Jiadi Weng**, **Jiani Zhang**, **Jizong Zhao**, **Xiaolin Chen**, and **Bin Dong**: writing – review and editing. **Fa Lin**, **Jian Lyu**, **Wenxiong Song**, **Bingcheng Zhu**, **Minghao Liu**, **Jionghao Xue**, and **Jiadi Weng**: visualization. **Jizong Zhao**, **Xiaolin Chen** and **Bin Dong**: supervision. **Fa Lin** and **Bin Dong**: funding acquisition. Fa Lin and Jian Lyu contributed equally to this work. All authors approved the manuscript.

## Conflicts of Interest

The authors declare no conflicts of interest.

## Data Availability

Data sharing is not applicable to this Review because no new data were generated or analyzed.
